# The impact of Metzembaum septoplasty on nasal and facial growth in children

**DOI:** 10.5935/1808-8694.20130081

**Published:** 2015-10-08

**Authors:** Denise Barreiro Costa, Wilma Terezinha Anselmo-Lima, Edwin Tamashiro, Carla Enoki, Fabiana Cardoso Pereira Valera

**Affiliations:** a Graduate student - Department of Ophthalmology, Otorhinolaryngology and Head and Neck Surgery, Medical School of Ribeirão Preto - University of São Paulo (FMRP-USP).; b Professor - Department of Ophthalmology, Otorhinolaryngology and Head and Neck Surgery, Medical School of Ribeirão Preto - University of São Paulo (FMRP-USP).; c Assistant Professor, Division of Orthodontics - Dentistry School of Ribeirão Preto - University of São Paulo.; d Post doctorate (PhD professor of FMRP - USP).; Faculty of Medicine of Ribeirão Preto - University of São Paulo.

**Keywords:** anthropometry, facial bones, growth, maxillofacial development, nasal septum, nasal septum/growth and development

## Abstract

Several studies have investigated the effects of septoplasty on facial growth in children, with conflicting results. However, just handful of those employed objective measures or evaluated patients after facial growth completion.

**Objective:**

This study assesses the effects of the Metzenbaum septoplasty, which preserves the perichondrium and growth-related areas on nasal and facial growth in children.

**Method:**

We included those children submitted to surgery before the age of 14 and who had 16 years or years of follow up. Sixteen patients were selected. We evaluated the following parameters: clinical satisfaction (nasal patency and aesthetics), anthropometric measurements and cephalometry. Scientific design: cross-sectional historical cohort.

**Results:**

The mean age at surgery was 13 years, children were assessed on average 4.3 years after surgery. Only one patient had anthropometric and cephalometric values below normal, but no aesthetics or patency complaints. Four other patients complained about their nasal aesthetics and three had patency complaints.

**Conclusion:**

The Metzenbaum septoplasty appears to be a safe technique to correct caudal septum deviations. This technique had no significant impact on facial growth of the patients assessed.

## INTRODUCTION

The surgical treatment of obstructive septal deformity in children and the time to perform surgical correction are still controversial issues. However, some authors^1^, ^2^, ^3^ have advocated early surgical correction in cases of obstructive nasal septum deformities, with the justification that the restoration of nasal breathing would lead to a normalization of the child's development as a whole, especially in the middle third of the face.

In general, septal deformities in children, usually located in its caudal portion, cause considerable symptoms because they involve the nasal valve area. It is known that caudal septal defects are a major cause of chronic nasal obstruction in children^4^, having the nasal trauma during birth as the most frequent cause, which prevalence ranges from 0.5 to 25%. Since such area is weakened, there is a tendency for the deviation to worsen with the subsequent facial growth^5^.

Several surgical techniques for septal deformity correction have been described in the literature^6^, ^7^, ^8^, all aiming at preserving as much as possible of the nasal growth areas and the mucoperichondrium, which seem to have a critical role in septal and nasal growth^9^.

Questions about the impact septoplasty has on nasal growth are frequent and experimental studies in animal models have shown different effects on nasal growth^10^, ^11^, ^12^. Although longitudinal studies already performed in humans show no midfacial growth retardation, few used objective measures of the face or evaluated patients after completion of their facial growth^1^, ^7^, ^13^, ^14^, ^15^.

The aim of this study was to evaluate the effects of Metzenbaum septoplasty done to children, on the nasal and facial growth in patients undergoing surgery while still within the facial growth phase and evaluated after growth completion.

## METHOD

We evaluated patients submitted to Metzenbaum septoplasty from January of 2000 through December of 2008, aged less than or equal to 14 years at the time of surgery, and at least 16 years at the time of the clinical and radiological assessment. We included only patients submitted to Metzenbaum septoplasty as the only surgical procedure in the septum, and two patients were additionally submitted to adenoidectomy, three to adenotonsillectomy and inferior turbinates linear cauterization at the same time of the septoplasty. We took off those patients submitted to another septal surgery after the initial procedure, patients with septal deformities other than the caudal defect, patients with genetic syndromes or other conditions that naturally altered facial proportions; and non-caucasian patients, because the normality values are only available for caucasians.

The surgeries were performed by the residents of the University Hospital of Ribeirão Preto, always under the supervision of two professors of our team; the surgical technique was strictly followed in all the procedures.

Of the 81 patients submitted to surgery in this period, 27 fit the inclusion and exclusion criteria, and their medical records were reviewed. These patients were invited to come to our clinic for a three-phase assessment: an interview - during which they were asked about their satisfaction with nasal patency and nasal shape; we obtained anthropometric measurements of the face using a caliper^16^, and cephalometry was carried out in a specialized laboratory. The following parameters were evaluated according to previously described methods:

### Anthropometry^7^, ^16^, ^17^ (Figure 1)

The following points were analyzed in the patient:

1. Nasal height (n-sn);
2. Nasal dorsum length (n-prn);
3. Nasal tip protrusion (sn-prn);
4. Columella length (sn-c’);
5. Nasal width (al-al);
6. Columella width (sn-'sn’);
7. Facial width (zy-zy);
8. Face height (n-gn); and
9. Upper face height (n-sto).

**Figure 1:**
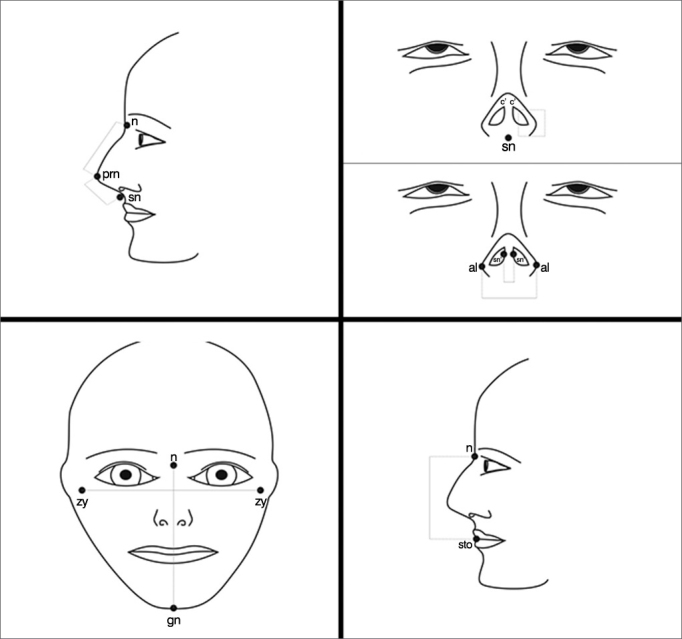
Anatomical parameters used for facial analysis. n: Nasion; prn: Pronasale; sn: Subnasale; c’: Columella apex; al: Nasal wing, most lateral point; sn’: Columella lateral border; zy: Zygion; gn: Gnation; sto: stomion^7^.

Four proportions were calculated from these measurements (Figure 1):

A) The ratio between nasal length and height (n-prn)/(n-Sn);
B) The ratio between columella length and nasal tip projection (sn-c’)/(sn-prn);
C) The ratio between nasal length and upper face length (sn-n)/(n-sto); and
D) The ratio between the height and width of the face, known as an the facial index.

### Cephalometry^18^, ^19^, ^20^, ^21^, ^22^, ^23^ (Figure 2)

Was asked patients to undergo a cephalometric profile, from which the following measurements were obtained:

1. Palatal length, from the anterior nasal spine (ANS) to the posterior nasal spine (PNS), in centimeters;
2. Protrusion of the midface (linear measurement), the sella (S) and the ANS;
3. Protrusion of the midface (angular measure), angle between the sella, nasion and the most concave point of the maxilla (A) (SNA);
4. Length of the midface - from the nasion (n) to the anterior nasal spine (ANS).

**Figure 2:**
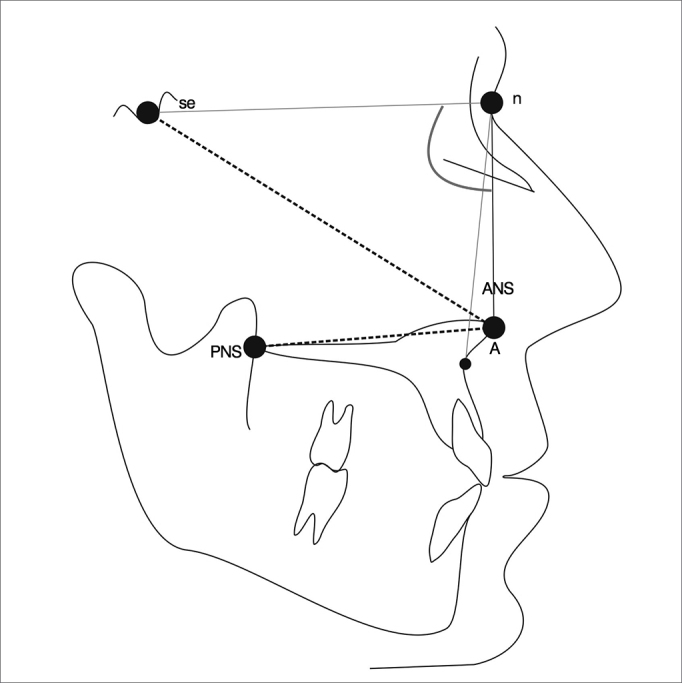
Cephalometric parameters used for facial analysis. S: Sella turcica; n: Nasion; ANS: Anterior nasal spine; PNS: Posterior nasal spine; A: Most concave point of the maxilla^23^.

The data from the anthropometric and cephalometric measurements were analyzed by comparison with normal values published in the international literature^7^, ^16^, ^17^, ^18^, ^19^, ^20^, ^21^, ^22^.

Normal values were those data within the mean ± 2 SD (standard deviation) range. Optimal parameters were those measures in the range of mean ± 1 SD. Values above the mean + 2 SD were considered as above the normal range and values below the average - 2 SD were considered as below normal.

This study was approved by the Ethics Committee in Research of the Medical School of Ribeirão Preto, under Protocol #4803/2010.

## RESULTS

Of the 27 patients, 16 (59%) returned to the Hospital for evaluation in the Department of Otolaryngology. Among these patients, 14 were males and two were females, with a mean age of 12.9 years (ranging between 10 and 14 years) on the day of surgery and 17 years (ranging between 16 and 20 years) on the date of evaluation. The mean postoperative follow up evaluation time of the patients was 4.3 years (ranging from 2 to 9.5).

During the interview, only one patient reported finding her nose small in relation to the size of the face. Four patients complained of nasal aesthetics, referring to the presence of a hump in the back or a wide nose. Three patients complained of intermittent nasal obstruction, although referring to an improvement in symptoms after surgery, with recurrence of obstruction in the late postoperative period. The others had no complaints.

**Figure 3:**
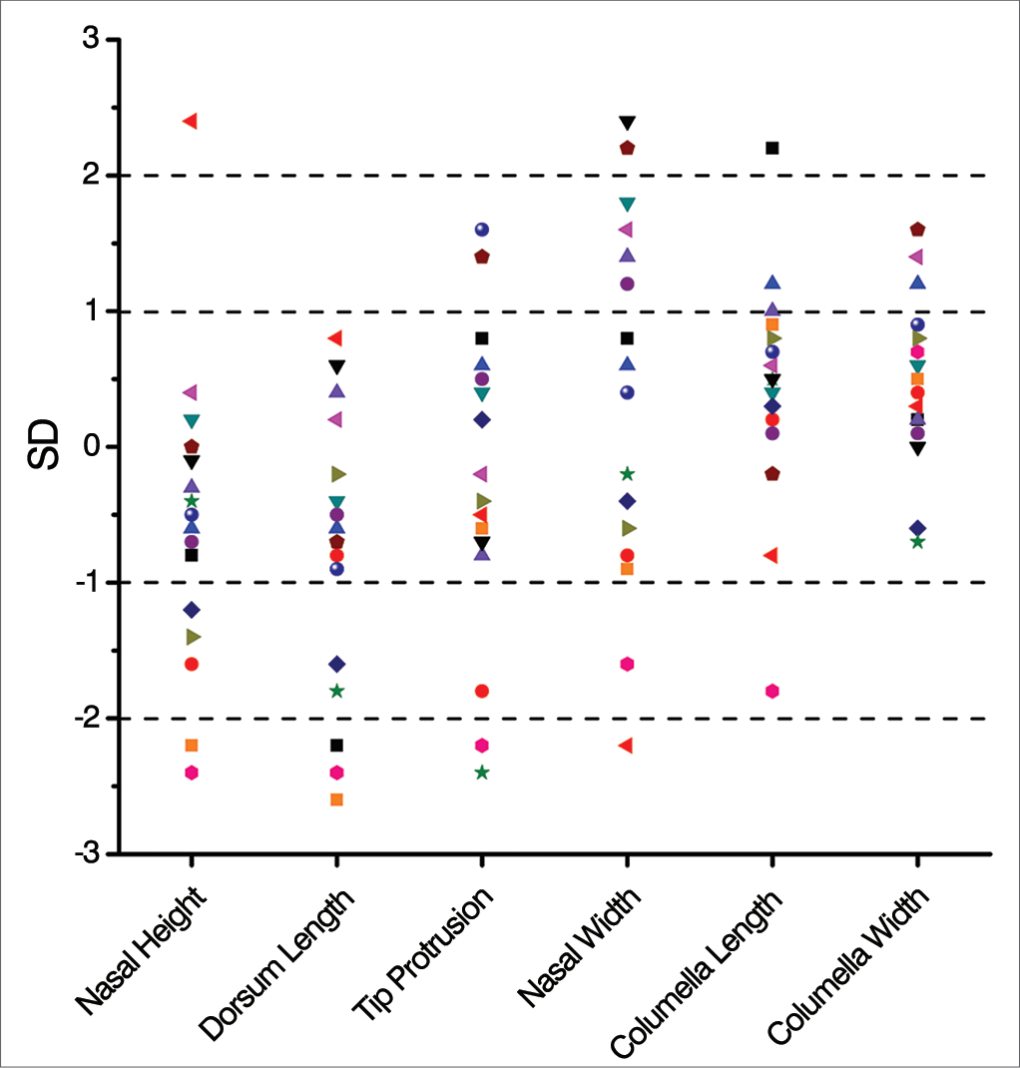
Mean and standard deviation distribution of the linear anthropometric measures in the late postoperative of patients submitted to the Metzenbaum septoplasty (n = 16).

Anthropometric data of nasal height and dorsum length, 13 patients (81.25%) had normal parameters, of which in 10 and 11 of them, respectively (62.25 and 68.75%), measures were considered optimal (Figure 3). For data relating to tip protrusion and nasal width, 14 patients (87.5%) had measurements within normal parameters, 8 and 13 of them, respectively (50% and 81.25%), had the measures considered optimal (Figure 3).

For data relating to the length of the columella, 15 patients (93.75%) had measures within normal limits, 13 of them (81.25%) were considered optimal (Figure 3). For data relating to the width of the columella, the 16 patients (100%) had measures within normal limits and 14 of them (87.5%) were considered optimal (Figure 3).

**Figure 4:**
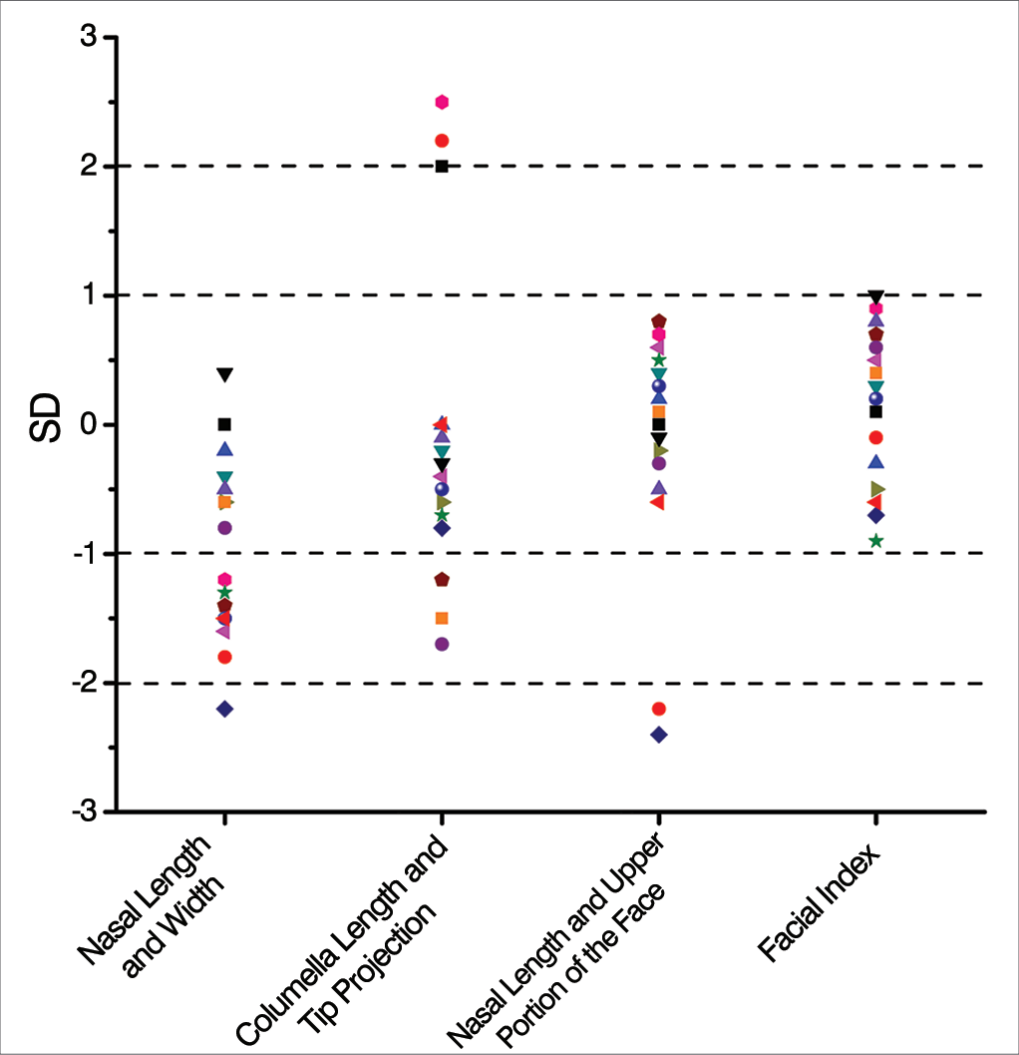
Mean and standard deviation distribution of the proportional late post-operative anthropometric measurements of patients submitted to Metzenbaum septoplasty (n = 16).

In relation to the facial proportional measures, 15 patients (93.75%) had the relationship between nasal length and height within normal limits, with eight of them (50%) with values were considered excellent (Figure 4). There were 13 normal measures of the relationship between the length of the columella and the nasal tip projection (81.25%), of which 10 (62.25%) were considered optimal (Figure 4).

The ratio between nasal length and the length of the upper face and facial index measurements had 14 and 16 measures, respectively (87.5% and 100%) within normal limits, and all these measures were considered optimal (Figure 4). The evaluation of facial proportions showed harmony among facial measurements, especially in the facial index, which was optimal in all cases.

**Figure 5:**
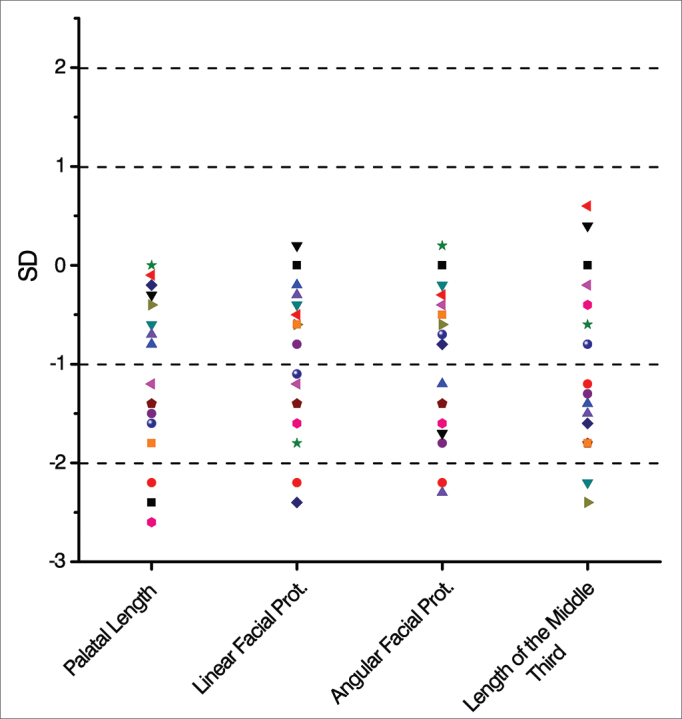
Mean and standard deviation distribution of the late postoperative cephalometric measurements of the patients submitted to Metzenbaum septoplasty (n = 16).

The cephalometric measurements were within the normal range in most cases; in relation to palatal length, 13 measures (81.25%) were normal, and eight (50%) of them were considered optimal (Figure 5). For cephalometric data concerning linear and angular facial protrusion and length of the middle third, 14 of the 16 measures (87.5%) were within normal limits. Nine and seven of the 16 measures (56.25% and 43.75%) of facial protrusion and length of the middle third, respectively, were considered optimal (Figure 5).

Of all patients analyzed, we found that only one had measures below normal anthropometric values (nasal height, dorsum length, tip protrusion) and cephalometry (palatal length). This individual did not complain of nasal aesthetics or function.

The patient who complained of having a small nose had measures below normal for the following parameters: nasal height and dorsum length. The remaining measurements of this individual were normal.

The other below-normal values happened at random, i.e. did not match to a particular individual, being distributed among all the patients in the study, without repeating the pattern.

## DISCUSSION

Metzenbaum Septoplasty is a surgery that involves removing deviated portions of the anterior quadrangular cartilage - responsible for much of the obstructive symptoms in patients during childhood. Despite the removal of a strip of cartilage, the mucoperichondrium is completely preserved during the procedure.

The literature shows controversial studies regarding facial growth when animals are submitted to septoplasty during the growth phase, even when the mucoperichondrium is preserved. Recently, Wong et al.^11^ reported changes in the facial growth of rabbits after septoplasty. However, Cupero et al.^10^ observed no such statistical difference in ferrets submitted to this surgery under the same circumstances.

In humans, numerous studies have tried to assess the negative effects of septoplasty on facial growth^9^. Although most of them are based on subjective measures of facial growth, most did not report any change in facial proportions^2^, ^15^.

It is known that values related to facial proportions are good predictors of appropriate facial growth^7^. Human studies that used objective measures did not seem to demonstrate influence on facial growth; they were, however, of short follow-up period. Walker et al.^6^ evaluated 10 children after external-approach septoplasty employed to correct deviations anterior to the nasal spine and observed no change in facial growth two years after surgery. El-Hakim et al.^23^ evaluated 26 children, using anthropometry before and after septoplasty, they showed no change in facial growth; however, the average age of the postoperative evaluation was 12.5 years, an age at which facial growth is still incomplete.

Studies show that nasal maturation occurs between 14 and 16 years for boys and between 12 and 14 years for girls^18^, ^24^. The evaluation performed in our study considered 16 patients who underwent a less invasive technique of septoplasty during puberty (between 10 and 14 years), with facial parameters evaluation only after completion of the facial growth period (between 16 and 20 years) as determined by Heijden et al.^18^ in their study on nasal growth and maturation in adolescents. According to these authors, traditional rhino-septal surgery can be safely performed in girls older than 16 years and in boys older than 17 years of age, a period in which there is a significant slowdown in facial growth.

The objective anthropometric and cephalometric measures obtained in our study were compared with normal values previously reported in the international literature. The results, in most cases, were within normal ranges for the age and gender of the patient being studied^7^, ^16^, ^17^, ^18^, ^19^, ^20^, ^21^, ^22^. The sample had only Caucasian patients, because we only have normality data for this population. Although the sample was small, the study is representative for being the only one with long follow up and with patients above 16 years of age at the last evaluation.

Among all parameters measured in this study, the majority lies within a range considered optimal, i.e., within the range of ± 1 SD from the mean. The most suited values were the facial index (100%) and the length of the columella (81.25%), followed by the other anthropometric measures.

Only one patient (6%) had measurements at the lower limit, or below normal for most of the anthropometric and cephalometric parameters being regarded as having a small nose to the face. This patient did not complain of nasal aesthetics or patency. Other patients had random alterations in their measurements, without an established pattern of change to a particular patient.

Thus, the Metzenbaum approach, even when performed in the period of facial growth, does not appear to influence nose development. This result is in agreement with a recent review paper published by Lawrence^25^, which concludes that there is now evidence that septoplasty, if done carefully, does not have a negative impact on nasal and facial growth.

## CONCLUSION

Surgical correction of caudal septal deviations by the Metzembaum approach seems to be safe, without significant effects on facial growth during puberty, as long as the mucoperichondrium and growth areas are preserved.
